# Thermodynamics of sustaining liquid water within rough icephobic surfaces to achieve ultra-low ice adhesion

**DOI:** 10.1038/s41598-018-36268-5

**Published:** 2019-01-22

**Authors:** Tom Y. Zhao, Paul R. Jones, Neelesh A. Patankar

**Affiliations:** Northwestern University, Department of Mechanical Engineering, 2145 Sheridan Road, Evanston, Illinois 60208 United States

## Abstract

The build-up of ice on aircraft, bridges, oil rigs, wind turbines, electrical lines, and other surfaces exposed to cold environments diminishes their safe and effective operation. To engineer robust surfaces that reduce ice adhesion, it is necessary to understand the physics of what makes a surface icephobic (“ice-hating”) as well as the relationship between icephobicity and ice adhesion. Here we elucidate the molecular origin of icephobicity based on ice-surface interactions and characterize the correlation between material icephobicity and liquid wettability. This fundamental understanding of icephobic characteristics enables us to propose a robust design for topologically textured, icephobic surfaces. The design identifies the critical confinement length scale to sustain liquid water (as opposed to ice) in between roughness features and can reduce the strength of ice adhesion by over a factor of twenty-seven compared to traditional hydrophobic surfaces. The reduction in ice adhesion is due to the metastability of liquid water; as ambient ice cleaves from the textured surface, liquid water leaves confinement and freezes – a process which takes the system from a local energy minimum to a global energy minimum. This phase transition generates a detachment force that actively propels ambient ice from the surface.

## Introduction

Ice formation presents a persistent problem in processes where structures are subject to cold conditions. Ice buildup on aircraft wings can cause a 25% increase in drag and 90% reduction in lift^1^, while ice accretion on wind turbines can decrease annual energy production by 17%^2^. Hence, there is a need for engineered surfaces that suppress ice formation and reduce ice adhesion.

Topologically textured superhydrophobic surfaces have been shown to reduce both ice nucleation and adhesion by enhancing liquid droplet roll-off and sustaining ambient ice on top of the texture^3^. However, surface defects and homogeneous nucleation can eventually trigger the formation of ice on the substrate^4^. Water can also condense and freeze or desublimate in between surface texture, leading to ice occupation of the roughness and significantly increasing ice adhesion to the material^5^. Lastly, de-icing cycles erode surface roughness and degrade the ice-repellency of the substrate^6^. A robust, icephobic design that minimizes ice adhesion to the surface must therefore ensure that ambient ice does not fill the space between surface texture by impalement or phase change, despite surface defects or damage.

De-icing a substrate has also been facilitated by introducing other species onto the surface. Liquid impregnated textured surfaces (LIS^7^) and slippery liquid-infused porous surfaces (SLIPS^8^) can decrease ice adhesion by introducing a lubricant that more favorably wets a textured surface than water. In these methods, the lubricant must be constantly replenished.

Surface science approaches have largely focused on rough hydrophobic surfaces with the expectation that such surfaces would also be icephobic. However, hydrophobic surfaces are liquid-water-hating, which may not necessarily imply ice-hating. A fundamental understanding of icephobic characteristics is missing. We address this gap.

Our approach to designing anti-icing surfaces is founded on texturing a surface so that it is energetically favorable for ambient ice to melt in between roughness. This ensures one of two cases will occur: either the ambient ice remains unimpaled by resting on top of the surface roughness, or the ambient ice coexists in thermodynamic equilibrium with liquid water confined between texture. This surface design reduces ice adhesion permanently, remains robust against defects or damage, and does not require consistent application of an external species such as oils, lubricants or salts.

In this work, heterogeneous nucleation theory in the form of the Gibbs-Thomson equation gives the confinement length scale for which liquid water is stabilized between surface texture as a function of material properties and operating conditions^9,10^. In this thermodynamic framework, a surface which can sustain liquid water as opposed to ice in confinement is characterized by having an ice-liquid contact angle greater than 90°. This provides a physical basis for defining the icephobicity of a material.

Molecular dynamics simulations are then used to examine the interface between the different water phases (ice, liquid, vapor) and a solid substrate to uncover the molecular origin of icephobicity. The relationship between the various intrinsic contact angles of a substrate shows that both hydrophobic and hydrophilic substrates exhibit icephobic properties and can be textured appropriately to reduce ice adhesion.

Although pores enclosed entirely inside the substrate have been simulated with molecular dynamics^11^, this study examines periodic arrays of pores and pillars at the interface between ambient ice and the material substrate. The confinement length scales for two-phase equilibrium predicted from classical nucleation theory are shown to agree closely with simulation results for both types of texture at the interface, suggesting the underlying theoretical predictions may be generally applicable. We further elucidate how liquid water confined in equilibrium within surface roughness impact the physics of ice adhesion through an analysis of free energy and non-equilibrium steered molecular dynamics. These procedures inform the rational design of textured, icephobic surfaces that reduce ice adhesion to a minimum.

The mechanism for the reduction in ice adhesion is found to be due to the metastability of liquid water. We demonstrate that for icephobic surfaces sustaining confined liquid water, initiating cleavage of ice adhering to the textured surface activates a persistent force propelling the adhered ice away from the surface. This is due to the change in free energy associated with the phase transition of the liquid water to ice as it leaves confinement. As a result, the strength of ice adhesion to the textured surface is decreased by over a factor of twenty-seven from that to a perfectly smooth hydrophobic surface. A robust, de-icing surface exhibiting strong resistance to defects and damage can be achieved by texturing an icephobic substrate with deep pores or tall, periodic features with spacing below the prescribed critical confinement length scale.

## Ice-Liquid Phase Equilibrium: Flat Surface

The critical confinement length scale below which liquid water is sustained in between surface texture can be determined from heterogeneous nucleation theory. The theory describes the change in free energy associated with the formation of an ice particle from ambient, liquid water near a solid surface.

The maximum change in free energy with respect to the change in radius of the ice particle $(\frac{d{\rm{\Delta }}G}{dr}=0)$ gives the critical energy barrier that must be overcome for ice nucleation to occur (see the “Heterogeneous Nucleation” section of the Supporting Information):

1 $${\rm{\Delta }}{G}_{crit}=\frac{16\pi {\sigma }_{IL}^{3}}{3{\rho }_{I}^{2}{\rm{\Delta }}{h}_{F}^{2}}{(\frac{{T}_{F}}{{T}_{F}-T})}^{2}g({\theta }_{IL}),$$

where Δ*h*_*F*_ is the enthalpy of fusion^12^, *T*_*F*_ the bulk freezing temperature of water associated with the saturation pressure, and *T* the temperature of the liquid water in thermal equilibrium with the ice particle. The subscripts *I*, *L*, *S* denote the ice, liquid, and substrate, respectively, such that *ρ*_*I*_ is the density of ice^13^ and *σ*_*IL*_ the ice-liquid surface energy. The term *g*(*θ*_*IL*_) (equation (S6)) is a function of the intrinsic ice-liquid contact angle *θ*_*IL*_ with the substrate surface. The critical radius of the ice particle corresponding to this energy barrier is:

2 $${r}_{crit}^{IL}=\frac{2{\sigma }_{IL}}{{\rho }_{I}{\rm{\Delta }}{h}_{F}}(\frac{1}{1-T/{T}_{F}}).$$

Ice particles with radii smaller than the critical value ($r < {r}_{crit}^{IL}$) will disperse and remain in the liquid state, as the free energy decreases with a reduction of the particle radius. Ice particles larger than the critical size ($r > {r}_{crit}^{IL}$) form nuclei that initiate crystal growth, as the free energy then decreases with increasing radius of the nuclei.

## Ice-Liquid Phase Equilibrium: Cylindrical Pore

For simplicity, the surface texture considered will be a cylindrical pore on an otherwise flat, icephobic surface (*θ*_*IL*_ > 90°) adjacent to bulk ice. The subsequent analysis assumes that the height of the pore is much larger than its radius. Although the cylindrical pore is not a typical anti-icing surface roughness explored in current experiments, it will be shown later that the critical pore radius derived from considering a cylindrical geometry provides a good estimate of the critical confinement length scale for general surface texture. In addition, the cylindrical geometry of the pore is a meaningful approximation for porous substrates; the present study will show that porous surfaces can demonstrate robust de-icing performance, motivating their use in future anti-icing experiments.

For the cylindrical pore to sustain liquid water in thermodynamic equilibrium with the ambient ice phase, the pore radius *R* must be smaller than a critical value ${R}_{crit}^{IL}$ to prevent the formation of solid nuclei in confinement that initiate growth of the ice phase. Geometric considerations for the largest possible ice particle existing in mechanical equilibrium in the cylindrical pore gives the Gibbs-Thomson equation for the critical pore radius^10^:

3 $${R}_{crit}^{IL}({\theta }_{IL},T)=-\,{r}_{crit}^{IL}\,\cos ({\theta }_{IL})=\,\cos ({\theta }_{IL})\frac{2{\sigma }_{IL}}{{\rho }_{I}{\rm{\Delta }}{h}_{F}}(\frac{1}{T/{T}_{F}-1})$$

where the contact angle *θ*_*IL*_ of the ice-liquid interface with the icephobic surface should be the equilibrium contact angle defined in equation (12). Therefore the confined phase is liquid if *R* satisfies:

4 $$R < -\,{r}_{crit}^{IL}\,\cos ({\theta }_{IL}\mathrm{)}.$$

The confined phase is ice otherwise. This criterion for the pore radius enforces thermodynamic equilibrium between the confined liquid phase and the ambient ice. Ice particles do not nucleate in the confined liquid water inside the pore, and ambient ice does not grow into the pore, instead forming a ice-liquid meniscus pinned at the top edge of the pore. This analysis is similar to that for liquid-vapor phase change presented earlier^14,15^.

${R}_{crit}^{IL}$ defines a critical confinement length scale informing the design of surface texture that passively sustains liquid water in between surface roughness as a function of the ambient temperature and pressure, as well as the intrinsic ice-liquid contact angle *θ*_*IL*_ of the substrate material. As will be demonstrated by molecular dynamics simulations, ${R}_{crit}^{IL}$ not only applies to general pore geometries, but also to pillar-type texture.

## Condensed Water-Vapor Phase Equilibrium: Cylindrical Pore

To explore other possible phase transition pathways that could result in ice filling the space between surface texture, we examine the coexistence condition between water vapor and condensed water (ice or liquid), which describes the condition for desublimation or condensation in confinement. The critical radius ${r}_{crit}^{CV}$ of the ice or liquid nucleus in its surrounding vapor is^15,16^:

5 $${r}_{crit}^{CV}=\frac{2{v}_{C}{\sigma }_{CV}}{\bar{R}T\,\mathrm{log}(\frac{{p}_{V}}{{p}_{S}})}$$

where the subscript *C* denotes the condensed phase (ice or liquid) and the subscript *V* denotes the vapor. $\bar{R}$ is the specific gas constant, *v*_*C*_ the specific volume, *p*_*V*_ the vapor pressure, and *p*_*S*_ the saturation vapor pressure at temperature *T* over the condensed phase. Mechanical equilibrium between the condensed phase and water vapor across a curved interface is described by the Young-Laplace equation:

6 $${r}_{crit}^{CV}=\frac{2{\sigma }_{CV}}{{p}_{V}-{p}_{C}}.$$

Analogous to equation (3), the critical pore radius ${R}_{crit}^{CV}$ to sustain confined vapor in a cylindrical pore is then given by:

7 $${R}_{crit}^{CV}=-\,{r}_{crit}^{CV}\,\cos ({\theta }_{CV}\mathrm{)}.$$

Therefore, the equilibrium confined phase is vapor if the pore radius $R < {R}_{crit}^{CV}$. The confined phase is liquid or ice otherwise. Typically, *p*_*V*_ (vapor pressure) or *p*_*C*_ is known depending on whether the ambient phase outside of the pore is vapor or condensed water, respectively.

**Figure 1:**
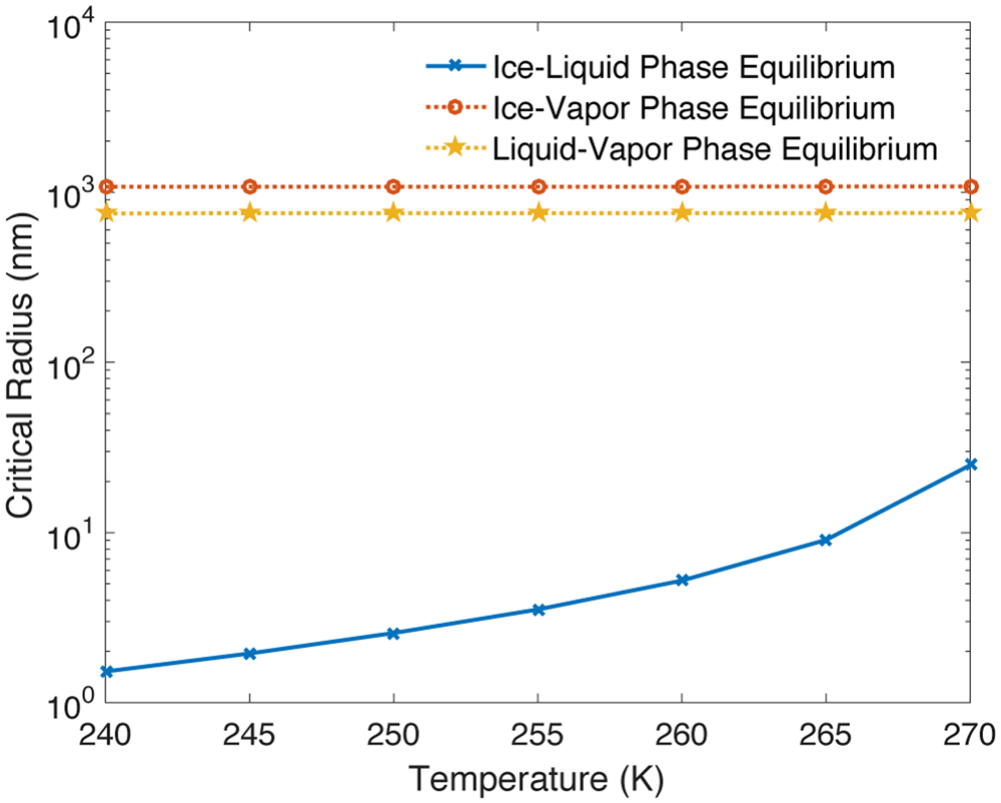
The critical pore radii (${R}_{crit}^{IL}$, ${R}_{crit}^{LV}$ and ${R}_{crit}^{IV}$) for phase equilibrium between liquid, ice or vapor at *p*_*C*_ = 1 atm as a function of temperature. The critical confinement length scale for freezing *R*_*IL*_ is the most restrictive of the three possible two-phase equilibria; a robust anti-icing surface must therefore satisfy *R* < *R*_*IL*_. To compare the order of magnitude values of the three critical confinement length scales, the material contact angles are taken to be the same for simplicity (*θ*_*LV*_ = *θ*_*IV*_ = *θ*_*IL*_ = 120°). The actual relationship among the intrinsic contact angles is established subsequently using molecular dynamics.

Figure 1 shows the dependence of ${R}_{crit}^{CV}$ and ${R}_{crit}^{IL}$ on temperature. For ${R}_{crit}^{CV}$, the ambient phase outside of the pore is assumed to be ice or liquid at *p*_*C*_ = 1 atm, and the confined phase is vapor; for ${R}_{crit}^{IL}$, the ambient phase is assumed to be ice, and the confined phase liquid. ${R}_{crit}^{CV}$ then describes the confinement length scale below which condensed water will not fill the space between the surface roughness through impalement or phase change. Indeed, Supplementary Fig. S3 uses molecular dynamics to verify that ambient ice or liquid initially resting on top of the surface texture will not fill the pore if its radius $R < {R}_{crit}^{CV}$, since the confined vapor and ambient condensed water outside the pore are in thermodynamic equilibrium. Note that a simplifying assumption is made to compare the order of magnitude values of the three critical confinement length scales: *θ*_*LV*_ = *θ*_*IV*_ = *θ*_*IL*_ = 120°. This does hold not true in general for a specific substrate; however, a relationship between these contact angles has not been established experimentally or computationally in the literature. The assumption is not made for subsequent analyses. Instead, this study will examine the relationship between the three intrinsic contact angles using molecular dynamics.

The critical confinement length scale for the reverse case, in which ambient vapor outside the pore exists in equilibrium with the confined condensed phase, is shown in Supplementary Fig. S4; this length scale is highly sensitive to the supersaturation of the ambient vapor.

The critical confinement length scale for freezing ${R}_{crit}^{IL}$ is the most restrictive of the possible pathways for confined ice to occupy the pore; any condensed phase that fills the pore from non-equilibrium events such as rain or cloud droplet impacts will freeze into confined ice if $R > {R}_{crit}^{IL}$. A robust design requires that the surface be icephobic and that the texture satisfy $R < {R}_{crit}^{IL}$ in order to prevent water from freezing in between surface roughness.

## Results and Discussion

### Icephobicity: Liquid Wettability and Molecular Origin

Due to the difficulty in measuring the ice-liquid contact angle experimentally, it is desirable to relate the intrinsic ice-liquid contact angle *θ*_*IL*_ (icephobic or icephilic) with the liquid-vapor contact angle *θ*_*LV*_ (hydrophobic or hydrophilic) of a substrate. Since both *θ*_*IL*_ and *θ*_*LV*_ are functions of temperature and pressure, the choice of ambient conditions may be somewhat arbitrary. We have chosen to compute *θ*_*IL*_ at *T* = 255 K and *p*_*C*_ = 1 atm, corresponding to the lower range of freezing depression induced by salts; and *θ*_*LV*_ at *T* = 300 K and *p*_*C*_ = 1 atm, corresponding to standard laboratory conditions. The contact angles were calculated from equation (12) by using molecular dynamics to determine the appropriate two-phase surface energies for a flat, planar interface^17^. This characterization of *θ*_*IL*_ offers insight into what makes a surface truly icephobic.

**Figure 2:**
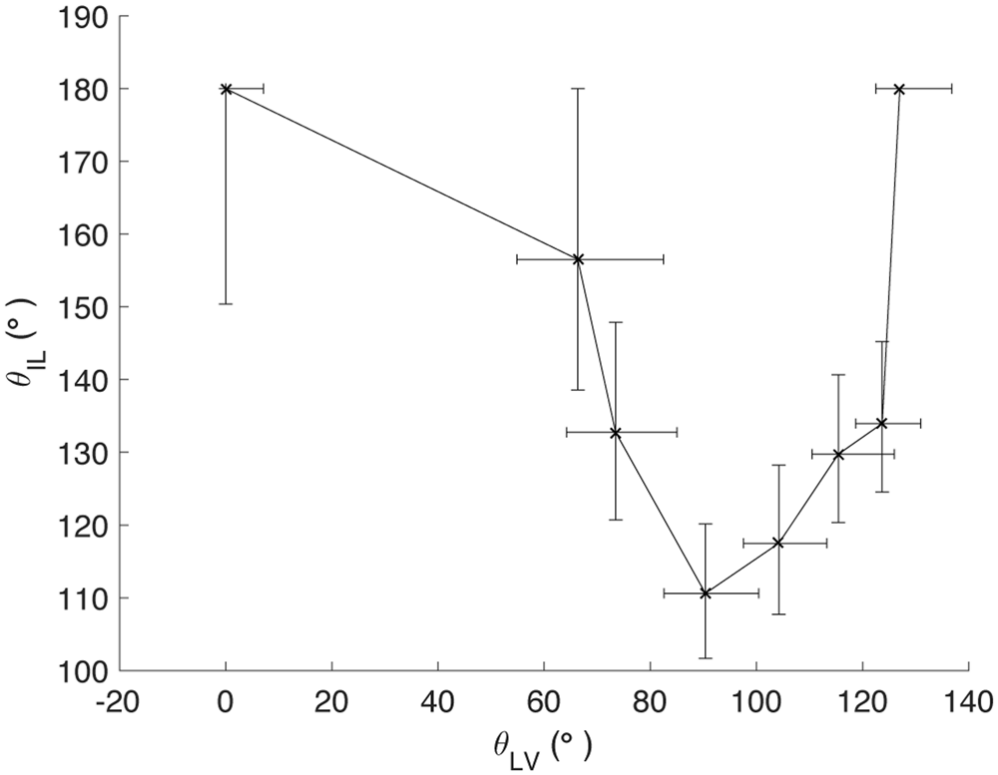
The intrinsic ice-liquid contact angle *θ*_*IL*_ of a material as a function of its intrinsic liquid-vapor contact angle *θ*_*LV*_. *θ*_*IL*_ is computed at *T* = 255 K, *p*_*C*_ = 1 atm, while *θ*_*LV*_ is computed at *T* = 300 K, *p*_*C*_ = 1 atm, both using equations (11 and 12). The error bars denote three standard errors.

Figure 2 shows from our simulations that both hydrophilic and hydrophobic materials are icephobic. From a minimum *θ*_*IL*_ > 90° at approximately neutral liquid wettability corresponding to *θ*_*LV*_ ≈ 90°, the intrinsic *θ*_*IL*_ increases both as *θ*_*LV*_ increases towards 180° and as *θ*_*LV*_ decreases to 0°. Supplementary Fig. S2 (**a**) shows that this icephobicity occurs due to the lower density of water in the contact layer (within 5 Å from the substrate surface) compared to bulk water density; water molecules favor positions in the bulk rather than at the interface with the solid substrate. This lower contact layer density penalizes the ice phase by deforming the lattice structure and introducing strain energy. On the other hand, the liquid phase exhibits significantly greater mobility in the contact layer compared to ice as indicated by the higher mean squared displacement <*u*^2^> of liquid water molecules (equation (S7)). This allows liquid water to achieve a lower, preferred interfacial density than the ice phase, even though the bulk density of liquid water is larger than that of ice. The favorability of liquid contact manifests in the ice-substrate surface energy exceeding the liquid-substrate surface energy for hydrophilic and hydrophobic materials, allowing both types of surfaces to sustain liquid water in between surface roughness. Similarly, Supplementary Fig. S1 shows that both hydrophobic and hydrophilic surfaces can sustain vapor in confinement since the intrinsic ice vapor contact angle *θ*_*IV*_ ≥ 90.

The non-monotonic dependence of *θ*_*IL*_ on *θ*_*LV*_ may occur in part due to the higher viscosity of interfacial liquid water on hydrophilic surfaces compared to hydrophobic surfaces^18^, which is corroborated by the drop in mobility of liquid water in the contact layer as *θ*_*LV*_ decreases (Supplementary Fig. S2). Li. *et al*. showed that this phenomenon increased the rate of ice nucleation on a hydrophobic surface by reducing the activation energy for diffusion of water molecules from the liquid to the ice phase. The change in both interfacial viscosity and density as *θ*_*LV*_ increases may result in a minimum difference in surface energy *σ*_*IS*_−*σ*_*LS*_ and a corresponding minimum in the ice-liquid contact angle *θ*_*IL*_ at neutral liquid wettability of the substrate.

### Icephobicity: Freezing and Melting Point Hysteresis

The choice between hydrophilic and hydrophobic substrates in anti-icing applications is settled by the need for robustness. Although both hydrophilic and hydrophobic surfaces can sustain liquid water in confinement, it is known that hydrophilic materials like silica support hysteresis between the melting and freezing temperatures^19^; however, such hysteresis has not been observed experimentally for hydrophobic materials.

Supplementary Fig. S5 shows that for hydrophilic substrates, the confined melting temperature is higher than the freezing temperature, whereas the confined melting and freezing temperatures are equal for hydrophobic substrates. In the former case, the metastability of the confined ice may be attributed to the hydrophilic substrate providing insufficient surface energy to overcome the volumetric energy barrier for melting; Supplementary Fig. S2 verifies that the surface energy of the substrate/ice interface is lower and the contact layer density higher for hydrophilic substrates compared to hydrophobic surfaces, such that the hydrophilic surface/ice interaction does not sufficiently perturb the local ice lattice to induce melting.

This phenomenon shows that the theoretically derived ${R}_{crit}^{IL}$ from binodal coexistence accurately describes the confinement freezing and melting temperature for hydrophobic substrates. Although classical nucleation theory can also estimate the confinement freezing temperature of hydrophilic substrates, capturing the confinement melting temperature requires analysis of the spinodal metastability of the ice phase due to the demonstrated hysteresis effect. Thus, the use of hydrophilic substrates in anti-icing applications necessitates quantification of the confinement phase change hysteresis as a function of operating conditions and surface hydrophilicity, which is out of the scope of the current study.

### Icephobicity: Critical Confinement Length Scale

**Figure 3:**
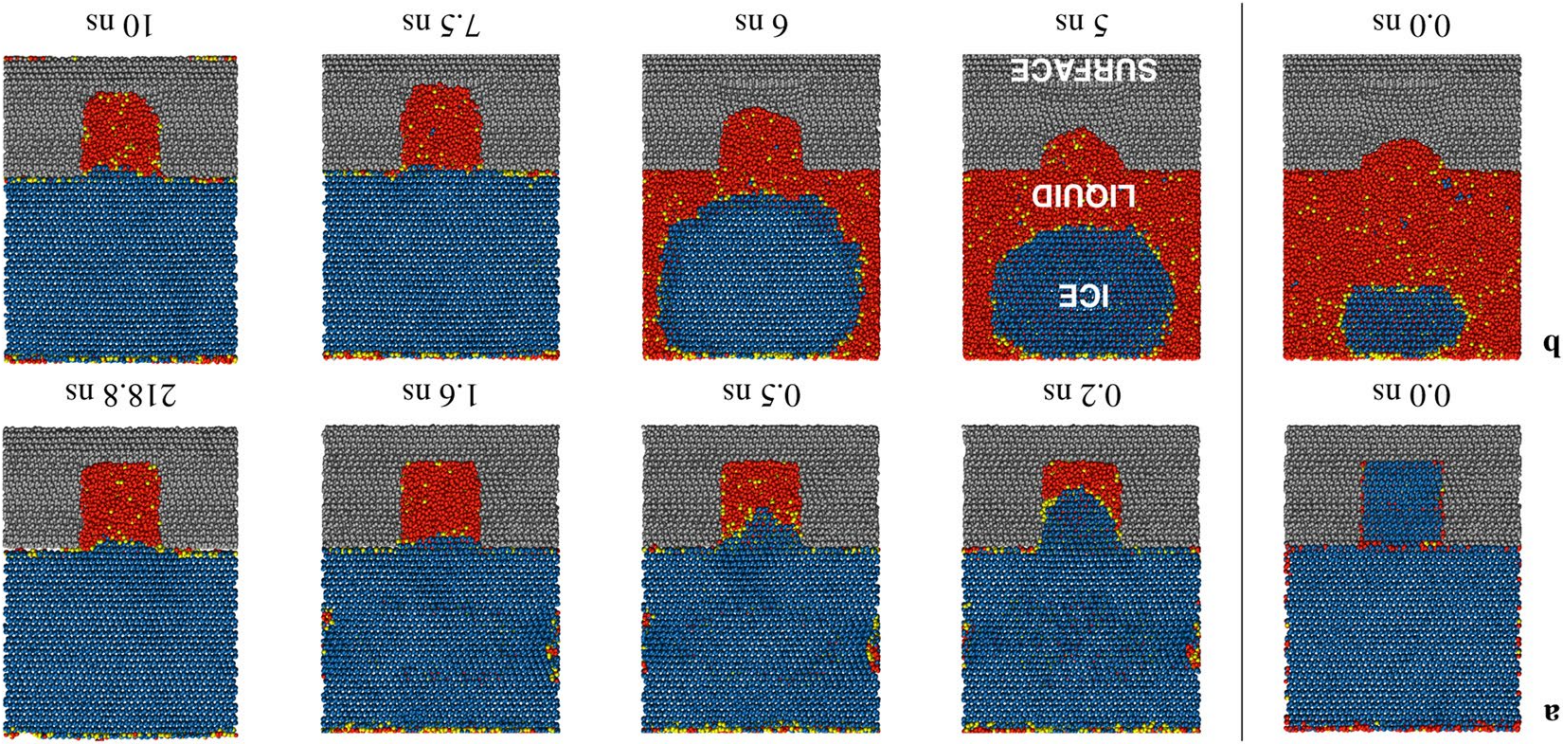
Molecular dynamics simulation of confined liquid water stabilized in a 5 nm diameter cylindrical pore on a substrate with *θ*_*LV*_ = 120.2° (computed at *T* = 300 K) and *θ*_*IL*_ = 134.0° (computed at *T* = 255 K). *T* = 255 K, and *p*_*C*_ = 1 atm. Cross-sections of the simulations are presented in **a** and **b**. In trajectory **a**, ambient ice (blue) is initially placed adjacent to a textured substrate (grey). In the cylindrical pore, the ice melts passively, producing amorphous glass (yellow) and liquid water (red) within the pore. In trajectory **b**, liquid water is initially placed adjacent to a textured substrate, where an ice nucleus is artificially introduced to initiate growth of the ice phase. The pore is initially empty with a liquid meniscus intruding from the bulk phase; the pore wall is curved compared to the adjacent substrate material. Inside the pore, the water does not freeze, instead remaining in the metastable liquid phase. Phases (lattice ice, amorphous glass, liquid) were colour coded using the CHILL algorithm^27^.

For icephobic substrates, molecular dynamics demonstrates that if $$R < {R}_{crit}^{IL}$$ is satisfied, the liquid phase can exist between surface texture in equilibrium with the ambient ice. Figure 3 shows two molecular dynamics simulations identifying the equilibrium phase of water confined in a cylindrical pore on an icephobic substrate. Cross-sections are visualized to clarify the three-dimensionality of the system. Temperature and pressure are held constant at 255 K and 1 atm, respectively. The radius of the pore is chosen such that *R* = 2.5 nm $$ < {R}_{crit}^{IL}$$.

Initially for trajectory (Fig. 3a), both the ambient and confined water are in the ice phase. Ambient ice on top of the surface remains frozen as time progresses, whereas ice inside the 5 nm diameter pore rapidly melts. Liquid water nucleates from the ice at the bottom of the pore and grows upward to the top of the cavity. The new state of the system persisted past 218 ns, the duration of the simulation.

Initially for trajectory (Fig. 3b), both the ambient and confined water are in the liquid phase, although an ice nucleus is artificially introduced to accelerate the dynamics; this models ice accretion initiated by homogeneous nucleation^4^. Ambient liquid on top of the surface freezes as time progresses, whereas water inside the 5 nm diameter pore remains in the liquid phase. The new state of the system persisted past 10 ns, the duration of the simulation.

The equilibrium coexistence states were sampled in molecular dynamics by continuing the simulation well past the point where the count of molecules in the ice and liquid phases remains constant with time (chemical equilibrium), the temperature of both phases remain constant and equal (thermal equilibrium), and the ice-liquid contact angle with the vertical pore wall is constant (mechanical equilibrium).

These trajectories along with Supplementary Fig. S3 show that if the critical confinement length scale is satisfied by the surface texture, the space in between texture will be occupied by either liquid water or vapor. There is no equilibrium state where ice is confined in between roughness.

For two substrate materials with different *θ*_*LV*_ and *θ*_*IL*_, Supplementary Table S1 and Fig. S7 show that the theoretical critical radii match well with simulation results. Molecular dynamics simulations also reveal that ${R}_{crit}^{IL}$ can accurately predict liquid wetting in surface texture as a function of the temperature (Supplementary Fig. S6). This agreement between theory and computation demonstrates the usefulness of ${R}_{crit}^{IL}$ in specifying the critical confinement length scale for surface texture as a function of the material icephobicity *θ*_*IL*_ and the operating conditions.

Due to the latent heat of fusion released (positive enthalpy difference) with phase change from liquid water to ice, the presence of the liquid water in between the surface texture should make this equilibrium state metastable. We show subsequently that sustaining liquid water in between surface texture corresponds to a local energy minimum. It can be further shown that this metastability is useful in reducing the strength of ice adhesion to the surface.

### Ice Adhesion: Metastability of the Liquid Water

We now wish to sample the energy landscape of the system in search of a possible global minimum away from the local minimum corresponding to confinement of liquid water in the pore. Metadynamics was used to reconstruct the free energy landscape in the space of a collective variable (CV) representing the vertical distance between the centers of mass of the ambient ice and the substrate^20^. The method uses a history dependent potential to bias the system away from local minima associated with particular values of the collective variable.

Note that the choice of the CV does not fully capture the complexity of de-icing pathways, which for instance can be expected to exhibit energy barriers in orthogonal shear directions or may involve some degree of interfacial premelting. Establishing a free energy landscape in the directions orthogonal to the ice/substrate interface necessitates the use of orthogonal CVs. Interfacial premelting occurs at low undercoolings and must be examined near the freezing point. Both analyses are outside the scope of this study.

Although the collective variable selected does not provide the most general description of possible ice/substrate dynamics, it does effectively describe the change in free energy associated with the removal of the ambient ice from the substrate. The free energy landscape in the vertical direction is used to probe the existence of local vs global energy minimia for porous surfaces, in contrast to the single global energy minimum expected for a flat substrate. Thus, the vertical distance between the centers of mass of the ambient ice and the underlying material is an appropriate choice for the collective variable in this context. The kinetics of ice detachment arising through shearing between the ambient ice and the substrate will be explored subsequently in this study using nonequilibrium simulations.

**Figure 4:**
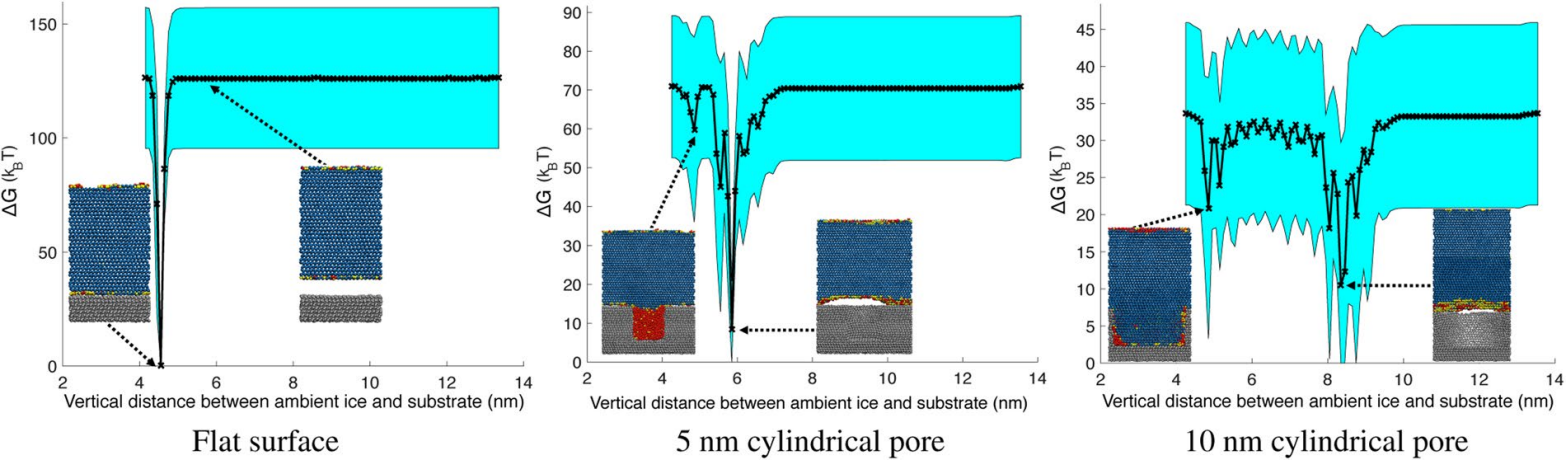
Change in free energy Δ*G* as a function of the vertical distance between the centers of mass of the ambient ice and the substrate. Cross-sections of the corresponding states in the molecular dynamics simulations are included for clarity. For a flat surface, there is a single global energy minimum for which ambient ice is attached to the surface. For porous surfaces, there are multiple local energy minima and a global energy minimum corresponding to ambient ice detached from the substrate and the initially confined phase evacuated from the pore. The wall of the empty pore associated with the global energy minimum is curved compared to the adjacent substrate material. *T* = 255 K, *p*_*C*_ = 1 atm, *θ*_*LV*_ = 120.2° (computed at *T* = 300 K), *θ*_*IL*_ = 134.0° (computed at *T* = 255 K). The heights of the pores are 7 nm. Metadynamics is carried out from 20 statistically independent initial conditions to reconstruct Δ*G*. The mean is the black curve and three standard errors is given by the cyan region.

Figure 4 shows the change in free energy as a function of the vertical distance between the centers of mass of the ambient ice and the substrate. The icephobic flat surface has a single, global energy minimum where the ambient ice is attached to the surface. To detach ambient ice from the substrate, a large external force must be applied to overcome the significant energy barrier in moving the system out of the global potential well into an unfavorable high energy state.

The 5 nm cylindrical pore satisfies $R < {R}_{crit}^{IL}$ and has a single, global energy minimum where the ambient ice phase has mostly detached from the icephobic substrate and the metastable liquid has frozen into ice after escaping the pore. The ambient ice adhering to the textured surface with the liquid water confined in the pore corresponds to a local energy minimum. To detach ambient ice from the substrate, a sufficient external force must be applied to overcome the small energy barrier separating the local energy minimum from the global potential well. As the ambient ice begins to cleave from the substrate, the liquid water adhering to the ambient ice escapes confinement and freezes. This initiated phase transition generates a force which drives the system down towards the global energy minimum, corresponding to the detached ice state.

Thus, the change in free energy elucidates the mechanism by which liquid water confined between surface texture reduces ice adhesion. Along with passively lubricating the interface, the liquid water confined in between surface texture actively does work in pushing ambient ice off the substrate as the metastable phase undergoes a freezing transition during the detachment process.

As the pore radius increases past the critical confinement length scale ($R\ge {R}_{crit}^{IL}$), the fraction of confined liquid water in the liquid phase decreases dramatically. For the 10 nm cylindrical pore where confined water is dominated by the ice phase, the activation energy to move the system out of the local energy minimum corresponding to attached ambient ice is much larger than for the case in which the confined water is liquid. Additionally, the numerous local minima in the path between the attached and detached states means a consistent force must be applied to overcome a series of energy barriers; the mechanical interlocking of the confined ice and surface texture hinders removal of the ambient ice.

For a hydrophobic substrate, the vapor phase is preferred inside the pore compared to the liquid phase (*σ*_*SV*_ < *σ*_*SL*_) from a surface energy perspective. One may thus be tempted to attribute the decrease in free energy associated with the detachment of the ambient ice from a porous substrate sustaining confined liquid water to this decrease in surface energy (*σ*_*SV*_ − *σ*_*SL*_ < 0). However, note that for an icephobic substrate, the liquid phase is preferred inside the pore compared to the ice phase (*σ*_*SL*_ < *σ*_*SI*_). Based on this surface energy analysis, the detachment of the ambient ice from the substrate with the 10 nm cylindrical pore should therefore demonstrate the largest decrease in free energy, since the initial confined phase inside the pore is ice (*σ*_*SV*_ − *σ*_*SI*_ < *σ*_*SV*_ − *σ*_*SL*_). However, this is not the case; the largest decrease in free energy occurs for porous substrates in which the confined phase is liquid. Thus, it is the phase transition of confined liquid water to ice that is responsible for producing the smallest energy barrier between the local (attached) and global (detached) energy minima for porous surfaces.

### Ice Adhesion: Strength of Ice Adhesion

The strength of ice adhesion measures the stability of the ice/substrate interface under shear, and is a practical engineering metric to quantify the de-icing capabilities of engineered surfaces. It can be measured in experiments^21^, which show that hydrophobic substrates with *θ*_*LV*_ ≈ 120 exhibit strength of ice adhesion around 160 kPa at −15 °C. Using molecular dynamics, ambient ice in each system was sheared from the substrate at a constant velocity using a harmonic spring. One end of the spring was attached to the center of mass of the ambient ice while the other end was attached to a reference point at an equilibrium distance from the ice. The reference point is then displaced at a constant velocity, so that the force applied to the bulk ice in the shear direction is proportional to the spring deflection from the equilibrium distance (equation (S8)). The shear force was processed through a Gaussian filter to remove the high frequencies attributed to thermal fluctuations and the resonance frequency of the spring^22^. The strength of ice adhesion was taken to be the maximum shear force at which ambient ice detaches from the surface divided by the projected interface area.

**Figure 5:**
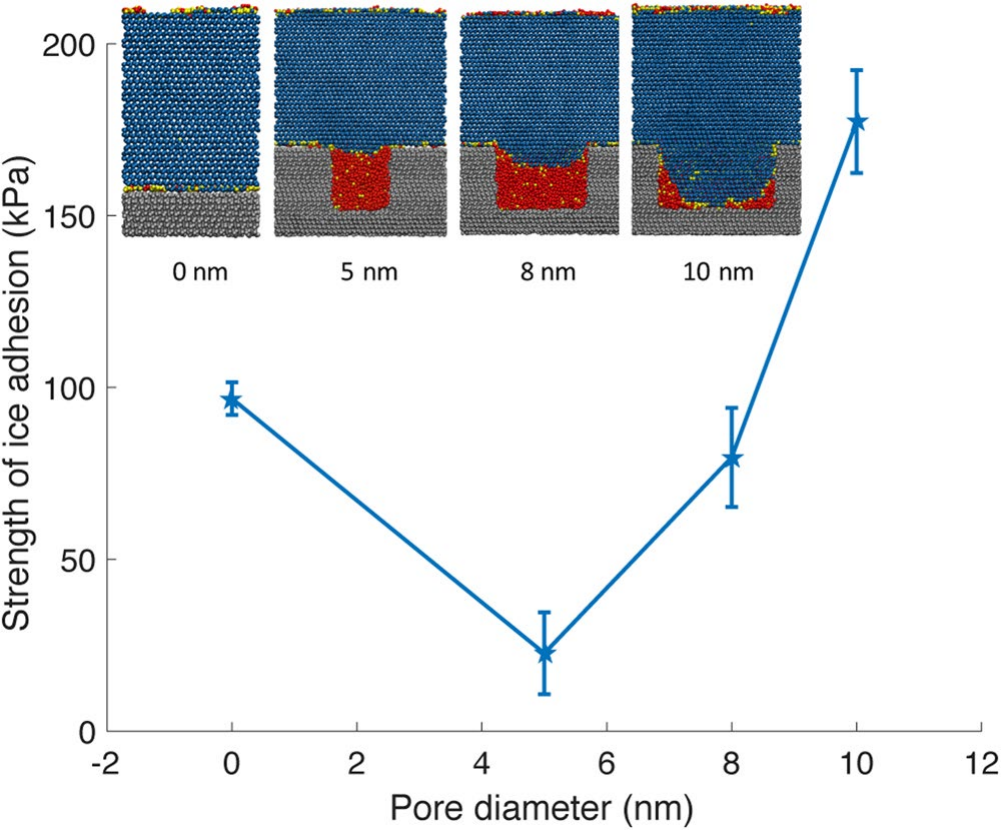
Strength of ice adhesion for cylindrical pores embedded in an icephobic substrate, compared with that for a flat surface (radius = 0 nm). *T* = 255 K, *p*_*C*_ = 1 atm, *θ*_*LV*_ = 120.2 (computed at *T* = 300 K), *θ*_*IL*_ = 134.0 (computed at *T* = 255 K). The error bars denote three standard errors. Cross-sections of molecular dynamics simulations with the corresponding pore diameters are included for clarity; the visualized snapshots correspond to equilibrium conditions before shear is applied.

Figure 5 shows that for the 5 nm diameter cylindrical pore in which the confined phase is completely dominated by liquid water, the strength of ice adhesion can be reduced by over a factor of four compared to that for the flat hydrophobic surface, which has roughness on the order of 2.8 Å. As the radius of the pore increases past ${R}_{crit}^{IL}$, the ice phase fills the pore. The mechanical interlocking between ice and the substrate for the 10 nm diameter pore leads to an increase in the strength of ice adhesion. Thus, textured surface can reduce or increase the strength of ice adhesion compared to a flat hydrophobic surface depending on whether the confined phase is liquid or ice, respectively.

The effect of pore geometry and hierarchical surface texture on the confinement length scale and the strength of ice adhesion is explored in Supplementary Figs S8, S9 and S10. It is shown that although selecting triangular prisms for the pore geometry can reduce the strength of ice adhesion compared to cylindrical pores, manipulating pore geometry or hierarchical structure does not significantly change the critical confinement length scale.

Supplementary Fig. S12 depicts the ice detachment pathways as a function of liquid wettability. For hydrophobic substrates (*θ*_*IL*_ > 90°) with roughness satisfying $R\le {R}_{crit}^{IL}$, the reduction in the strength of ice adhesion (Fig. 5) occurs when ice detachment is accompanied by dewetting of the texture. Since the ambient ice is effectively hydrophilic, the confined liquid will adhere to the ice rather than the hydrophobic texture during detachment. This de-icing pathway is therefore robust, and the reduction of ice adhesion will occur reproducibly and generically for hydrophobic texture satisfying the critical confinement length scale.

For hydrophilic substrates (*θ*_*IL*_ < 90°), the texture may not necessarily dewet during ice detachment. Supplementary Fig. S11 shows that lubrication from liquid water confined in hydrophilic roughness satisfying $R\le {R}_{crit}^{IL}$ can also reduce the strength of ice adhesion compared to that of a flat surface. For the roughness regime satisfying $R\,\gtrapprox \,{R}_{crit}^{IL}$, Supplementary Fig. S12 shows that the shearing of the ambient ice induces melting of the confined ice near the walls of the pore; this local phase change causes a change in surface free energy which decreases ice adhesion to a greater extent than can be achieved with lubrication effects. For both the phase change pathway and the lubrication pathway, the confined water does not escape during de-icing. Thus, there is a fundamental difference between the ice detachment pathways for hydrophobic and hydrophilic substrates sustaining a confined phase.

### Ice Adhesion: Periodic Surface Texture

**Figure 6:**
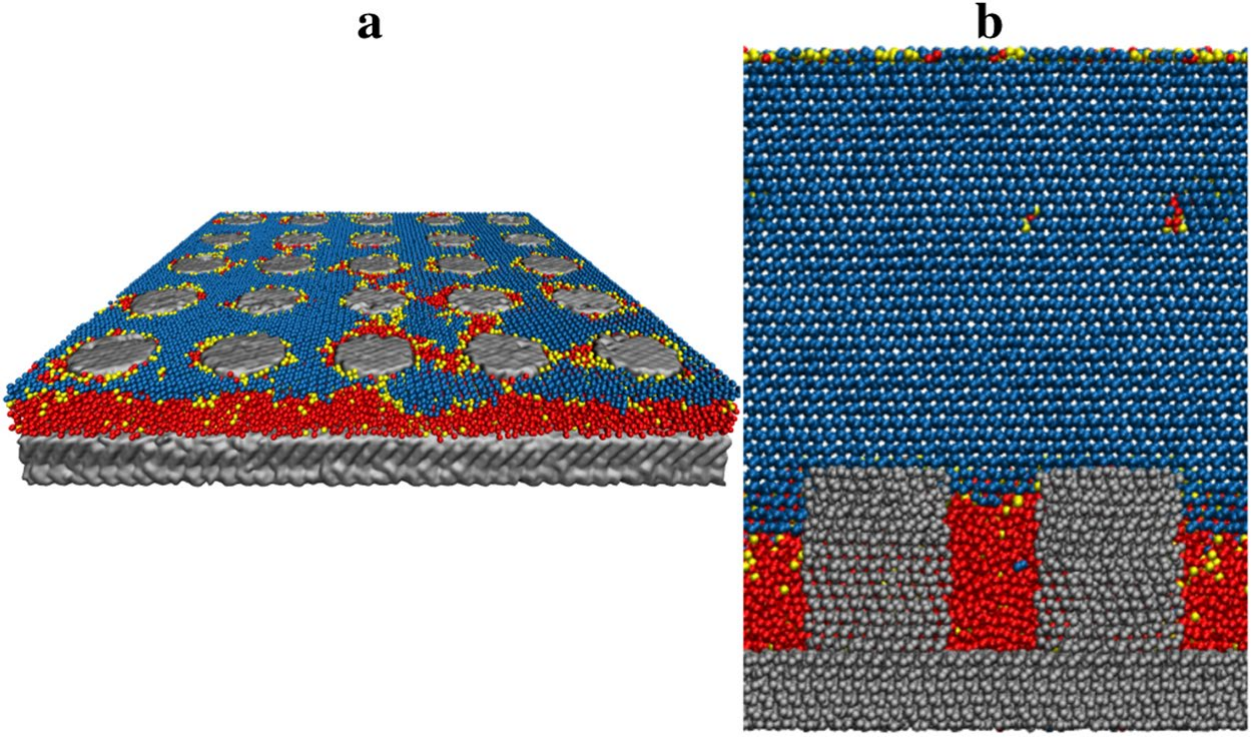
(**a**) Periodic array of cylindrical nanopillars arranged in a 5 by 5 grid. The radius of each pillar in this particular array is 2 nm, the spacing is 2.5 nm (defined as the radius of the inscribed circle in the projected area between four adjacent pillars). (**b**) A cross-sectional view of two adjacent nanopillars and the confined liquid water in the space between texture. *T* = 255 K, *p*_*C*_ = 1 atm, *θ*_*LV*_ = 120.2° (computed at *T* = 300 K), *θ*_*IL*_ = 134.0° (computed at *T* = 255 K).

The critical confinement length scale derived theoretically for cylindrical pores also applies reasonably for periodic arrays of icephobic, cylindrical pillars arranged on a substrate (Fig. 6). These periodic arrays can sustain a continuous film of liquid water, for which confinement is dictated by the spacing between roughness features.

**Figure 7:**
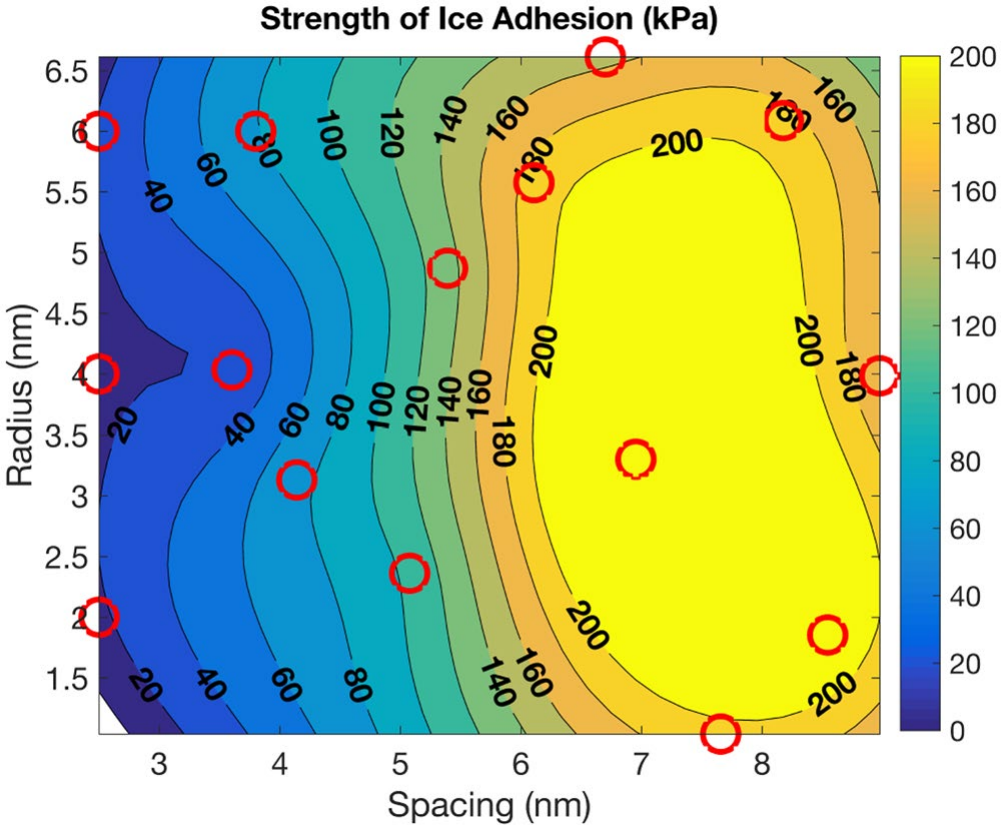
Strength of ice adhesion vs. radius and spacing (defined as the radius of the inscribed circle in the projected area between four adjacent pillars) of pillars in a periodic array. The height of each pillar is 2 nm. *T* = 255 K, *p*_*C*_ = 1 atm, *θ*_*LV*_ = 120.2° (computed at *T* = 300 K), *θ*_*IL*_ = 134.0° (computed at *T* = 255 K). The strength of ice adhesion for a flat surface is 96.75 kPa. The red circles correspond to simulation results; design of experiments was carried out using latin hypercubes. The exact strength of adhesion values are shown in Supplementary Fig. S13.

Figure 7 shows the strength of ice adhesion as a function of the spacing and radius of the pillars in the periodic lattice. The spacing is defined as the radius of the inscribed circle in the projected area between four adjacent pillars. The strength of ice adhesion on a flat substrate with *θ*_*LV*_ = 120.2° (computed at *T* = 300 K) and *θ*_*IL*_ = 134.0° (computed at *T* = 255 K) at *T* = 255 K and *p*_*C*_ = 1 atm is 96.75 kPa, which is on the same order of magnitude as experimental values^21^. The strength of ice adhesion for the pillared surface is less than for the flat surface when the pillar spacing ≲4.5 nm, which matches well with theory (${R}_{crit}^{IL}=3.8\pm 0.7$ nm). Note from the isocontours that the strength of ice adhesion depends mainly on the spacing between pillars (confinement length scale) and is less sensitive to the pillar radius. Thus, ${R}_{crit}^{IL}$ yields a general confinement length scale for the design of textured surfaces which can sustain liquid water in order to reduce ice adhesion.

**Figure 8:**
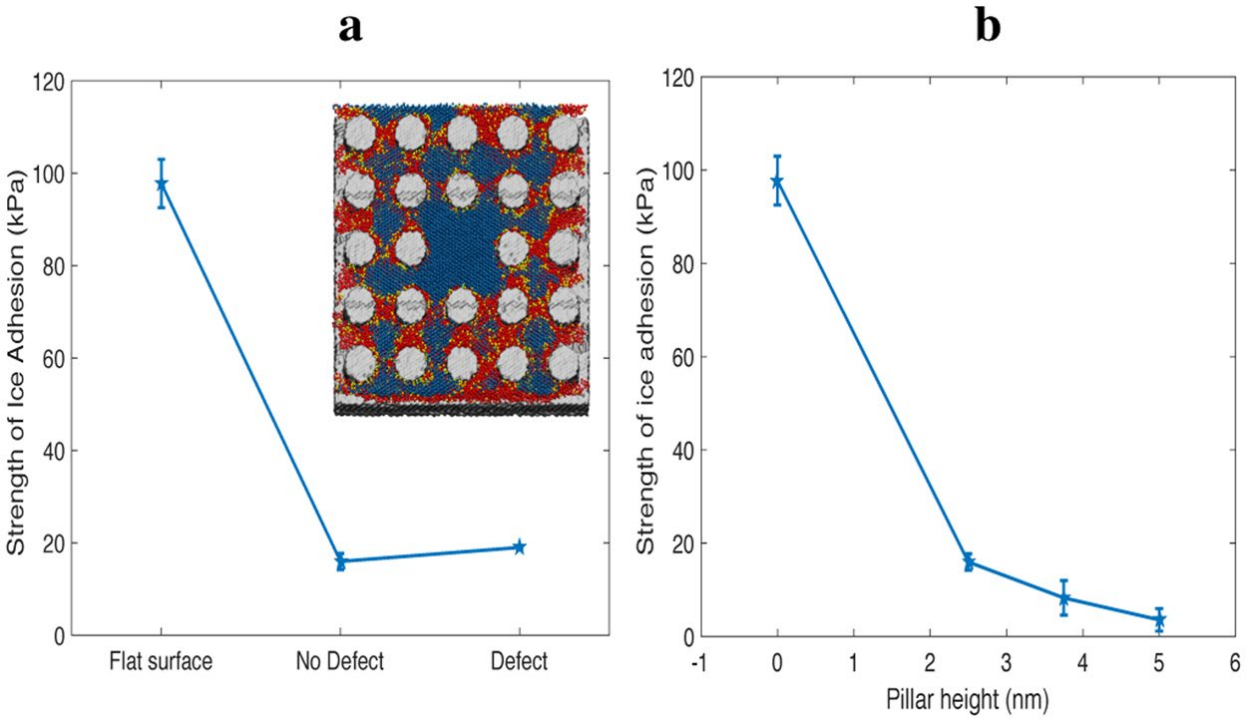
Strength of ice adhesion for pillars with and without defect (**a**) and as a function of pillar height (**b**). *T* = 255 K, *p*_*C*_ = 1 atm, *θ*_*LV*_ = 120.2° (computed at *T* = 300 K), *θ*_*IL*_ = 134.0° (computed at *T* = 255 K). The error bars denote three standard errors. A top down cross-section of the molecular dynamics simulation with a defect is included for clarity; the visualized snapshot corresponds to equilibrium conditions before shear is applied.

The anti-icing performance of these surface textures is also robust to local defects. Figure 8(a) shows a periodic array of pillars where one pillar is removed from the center of the lattice, introducing a defect that allows ambient ice to intrude into the liquid film. However, due to the abundance of liquid water in adjacent period cells, the increase in the strength of ice adhesion is negligible.

As the height of the pillars increases, the strength of ice adhesion decreases due to the increase in the amount of liquid water confined in between surface texture, as demonstrated in Fig. 8(b). Thus, periodic surface texture with the proper confinement length scale and sufficient depth can reduce the strength of adhesion by over a factor of twenty-seven compared to that for the flat hydrophobic surface. Since the strength of ice adhesion increases gradually as the height of the pillars decreases until the flat interface case is reached, the surface texture will continue to lower ice adhesion until it is completely destroyed. It is therefore desirable to engineer surfaces with tall texture or deep pores.

## Conclusions

In this work, material icephobicity is physically characterized by the ice-liquid contact angle satisfying *θ*_*IL*_ > 90°. The molecular origin of icephobicity arises from the rigid structure and reduced mobility of ice compared to liquid water at the interface with a material substrate, which results in a higher ice-substrate surface energy relative to the liquid-substrate interface. With this physical understanding and quantification of icephobicity, substrates may be appropriately engineered to reduce ice-adhesion to an icephobic surface.

The critical confinement radius below which liquid water exists in thermodynamic equilibrium with the adjacent bulk ice phase can be derived theoretically as a function of the ambient conditions (temperature, pressure) and surface wettability. The confinement of metastable liquid in between surface texture corresponds to a local energy minimum; as the ambient ice is sheared from the substrate, the liquid water escapes confinement and freezes, generating a detachment force driving the system to a global energy minimum where the ambient ice is mostly detached from the substrate. Icephobic surfaces textured according to the critical confinement length scale therefore undergo a permanent decrease in ice adhesion, either by the above mechanism or by ambient ice remaining suspended atop the surface roughness in the unimpaled state.

Surfaces that sustain liquid water in confinement are robust against local defects, which negligibly affects the global de-icing performance. As long as the confinement length scale is satisfied on average by the surface texture, the surface will continue to reduce ice adhesion until the texture is completely depleted. Porous or periodically textured surfaces are prime candidates for de-icing applications.

Materials with such properties include mesoporous zeolites and MOFs. A zeolite such as MCM-41 has pore diameters between 1 and 10 nm^23^; silylated MCM-41 has a liquid vapor contact angle of 133°^24^. Similarly, metallic organic frameworks such as MOF 5 have been polymerized to achieve liquid vapor contact angles of 135°^25^, and carbonized to achieve pore diameters around 6 nm^26^. MOF pores are interconnected, but as the periodic surface textures show, a continuous film of liquid water can be sustained between surface texture such that the ice/water interface area exceeds the ice/substrate interface area. Such appropriately textured, icephobic materials may be applied as robust surface coatings in a wide range of anti-icing and de-icing applications.

## Computational Methods

In this work, molecular dynamics is used to provide a quantitative characterization of material icephobicity and to probe the utility of liquid water sustained between surface roughness in reducing ice adhesion.

### Force Fields

The course-grained mW^17^ water model was used for computational efficiency and for precision capture of the hexagonal, cubic, and amorphous phases of ice. mW, proposed by Moore and Molerino, is a single particle model that accurately reproduces the structure, energetics, and phase transitions of water. The model comprises a re-parameterization of the Stillinger Webber potential, which incorporates a three-body term that penalizes non-tetrahedral configurations^17^:

8 $$E=\sum _{i}\sum _{j > i}\,{\varphi }_{2}({r}_{ij})+\sum _{i}\sum _{j > i}\,{\varphi }_{3}({r}_{ij},{r}_{ik},{\theta }_{ij})$$

9 $${\varphi }_{2}(r)=A\varepsilon [B{(\frac{\sigma }{r})}^{p}-{(\frac{\sigma }{r})}^{q}]\exp (\frac{\sigma }{r-a\sigma })$$

10 $${\varphi }_{3}(r,s,\theta )=\lambda \varepsilon {[\cos \theta -\cos {\theta }_{o}]}^{2}\exp (\frac{\gamma \sigma }{r-a\sigma }+\frac{\gamma \sigma }{s-a\sigma })$$

Ice nucleation and liquid diffusion are several times faster in mW than in water due to the absence of hydrogen atoms and long-range electrostatic calculations allows crystallization studies using mW to access longer time and length scales^27^.

### Molecular Dynamics

Molecular dynamics simulations were implemented using LAMMPS^28^ software. A periodic domain of water was initially crystallized at *T* < 273.15 K and *p*_*C*_ = 1 atm. A solid substrate material was constructed from mW molecules in the ice phase, similar to previous studies^27^. Molecules defining the substrate were tethered to their equilibrium positions. The interaction between the substrate and water was governed by a 6–12 Lennard Jones pair potential^11^; water-water as well as surface-surface interactions were governed by the three-body mW potential.

### CHILL Algorithm

The CHILL algorithm developed by Moore *et al*. differentiates cubic, hexagonal, and interfacial ice as well as liquid water and low density glass (amorphous ice). The local structures of the four closest neighbors of a mW molecule are projected onto a basis of spherical harmonics. The alignment of the local structure centered on each neighbor characterizes the central mW molecule as belonging to a specific ice or liquid phase^27^.

### Surface Energy

The surface energies *σ* between the various phase interfaces were calculated using the stress tensor method^17^ in molecular dynamics simulations:

11 $$\sigma ={L}_{z}({P}_{N}-{\bar{P}}_{T})$$

where *L*_*z*_ is the length of the simulation domain normal to the interface, *P*_*N*_ = *P*_*zz*_ is the normal component of the stress tensor with respect to the interface, and ${\bar{P}}_{T}=\frac{1}{2}({P}_{xx}+{P}_{yy})$ is the average of the tangential components of the stress tensor. From equation (12), the ratio of surface energies can be used to find the intrinsic, equilibrium contact angle for liquid-vapor, ice-liquid, and ice-vapor (*θ*_*LV*_, *θ*_*IL*_, *θ*_*IV*_) interaction with the substrate:

12 $$\cos ({\theta }_{IL})=\frac{{\sigma }_{LS}-{\sigma }_{IS}}{{\sigma }_{IL}}.$$

The surface energy between water and the substrate is changed to modulate surface hydrophobicity/icephobicity by altering the Lennard Jones energy parameter^11^. It would be of interest, but not in the scope of the current study, to examine how surface energy changes with surface polarity.

## Electronic supplementary material

Supplementary Information

## Data Availability

Data generated or analysed during this study are included in this published article (and its supplementary information files).
