# Supplementary material for: Thermodynamics of sustaining liquid water within rough icephobic surfaces to achieve ultra-low ice adhesion
Source: Sci Rep. 2019 Jan 22;9:258. doi: 10.1038/s41598-018-36268-5 (PMC6342967; doi:10.1038/s41598-018-36268-5)
Supplement: Supplementary file 1 — Supplementary Information [file 41598_2018_36268_MOESM1_ESM.pdf]

Supplementary Information: Thermodynamics of sustaining liquid water within rough icephobic surfaces to achieve ultra-low ice adhesion

Tom Y. Zhao[1,+], Paul R. Jones[1,+], and Neelesh A. Patankar[1,*]

[1]Northwestern University, Department of Mechanical Engineering: 2145 Sheridan Road, Evanston, Illinois 60208, United States
[*]n-patankar@northwestern.edu
[+]these authors contributed equally to this work

ABSTRACT

The build-up of ice on aircraft, bridges, oil rigs, wind turbines, electrical lines, and other surfaces exposed to cold environments diminishes their safe and effective operation. To engineer robust surfaces that reduce ice adhesion, it is necessary to understand the physics of what makes a surface icephobic ("ice-hating") as well as the relationship between icephobicity and ice adhesion. Here we elucidate the molecular origin of icephobicity based on ice-surface interactions and characterize the correlation between material icephobicity and liquid wettability. This fundamental understanding of icephobic characteristics enables us to propose a robust design for topologically textured, icephobic surfaces. The design identifies the critical confinement length scale to sustain liquid water (as opposed to ice) in between roughness features and can reduce the strength of ice adhesion by over a factor of twenty-seven compared to traditional hydrophobic surfaces. The reduction in ice adhesion is due to the metastability of liquid water; as ambient ice cleaves from the textured surface, liquid water leaves confinement and freezes – a process which takes the system from a local energy minimum to a global energy minimum. This phase transition generates a detachment force that actively propels ambient ice from the surface.

Supplementary Information (SI)

Heterogeneous Nucleation At constant temperature and pressure, the change in free energy governs the solidification process[1]:

$$\Delta G = -\rho_I V_I \Delta\mu_{LI} + A_{IL}\sigma_{IL} + A_{IS}(\sigma_{IS} - \sigma_{LS}), \tag{S1}$$

where subscripts I, L, S denote the ice, liquid, and substrate. ρ_I is the density of ice, V_I the volume occupied by the ice particle, $\Delta\mu_{LI}$ the difference in chemical potential between the liquid and ice phases, A the interfacial areas and σ the corresponding surface energies. The first term captures the change in volumetric free energy associated with phase-change from liquid to solid. The second and third terms represent the creation of new ice/liquid and ice/substrate interfaces and the destruction of the liquid/substrate interface as the ice particle forms on the flat surface.

Following equation (S1), the equilibrium contact angle θ_{IL} of the ice-liquid interface with the substrate surface can be determined by the balance of the surface energies in equation (12). Substituting for $\cos(\theta_{IL})$ yields:

$$\Delta G = -\rho V \Delta\mu_{LI} + \sigma_{IL}(A_{IL} - A_{IS}\cos(\theta_{IL})). \tag{S2}$$

Assuming that ice forms on the surface in a spherical shape with radius of curvature r, we have $V = \frac{\pi}{3}(1-\cos(\theta_{IL}))^2(2+\cos(\theta_{IL}))$, $A_{IL} = 2\pi r^2(1-\cos(\theta_{IL}))$, and $A_{IS} = \pi r^2(1-\cos(\theta_{IL})^2)$. The change in free energy associated with the formation of an ice particle with radius r is:

$$\Delta G = \left(-\rho\frac{4\pi r^3}{3}\Delta\mu_{LI} + \sigma_{IL}4\pi r^2\right)\left(\frac{1}{4}\cos(\theta_{IL})^3 - \frac{3}{4}\cos(\theta_{IL}) + \frac{1}{2}\right). \tag{S3}$$

The difference in chemical potential can be approximated as a function of temperature assuming that the heat capacity of the liquid and solid phase are near equal:

$$\Delta\mu_{LI} = \Delta h_F\left(\frac{T_F - T}{T_F}\right) \tag{S4}$$

where Δh_F is the enthalpy of fusion. Here, T_F is the bulk freezing point of water associated with the ambient pressure, and T is the temperature of the liquid water in thermal equilibrium with the ice particle. The change in free energy associated with the formation of an ice particle with radius r is:

$$\Delta G = \left(-\rho\frac{4\pi r^3}{3}\Delta h_F\left(\frac{T_F - T}{T_F}\right) + \sigma_{IL}4\pi r^2\right)g(\theta_{IL}) \tag{S5}$$

where we have collected the terms

$$g(\theta_{IL}) = \frac{1}{4}\cos(\theta_{IL})^3 - \frac{3}{4}\cos(\theta_{IL}) + \frac{1}{2} \tag{S6}$$

Intrinsic Water Contact Angles Figure S1 shows that the intrinsic ice-vapor contact angle $\theta_{IV} \geq 90°$ for hydrophilic and hydrophobic surfaces (θ_{LV} in the range $[0°\ 180°]$). Figure S2 (**b**) demonstrates that unlike the behavior of condensed water, the vapor density in the contact layer is slightly larger than the bulk density of vapor, due to the weak attraction between vapor molecules. The vapor contact layer density and the vapor-substrate surface tension are also relatively insensitive to different liquid wettabilities θ_{IL} of the substrate; nonetheless, both are lower than that for ice. However, as θ_{LV} decreases, the ice-substrate surface energy approaches the vapor-substrate surface energy. For hydrophilic substrates, the lattice strain decreases as the contact layer density of ice approaches the bulk value, but the vapor density in the contact layer is not responsive to the increased substrate/water attraction.

The mobility of the phase in the contact layer is calculated by

$$< u^2 > = < \frac{1}{dt}(r(t+dt) - r(t))^2 > \tag{S7}$$

where $r(t)$ is the position of a molecule as a function of time, dt the elapsed time, and the brackets denote averaging over all molecules in the contact layer.

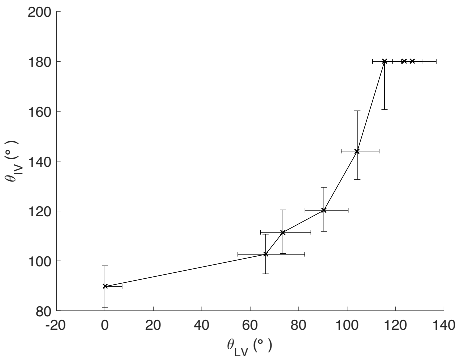

Figure S1. The intrinsic ice-vapor contact angle θ_{IV} of a material computed at $T = 255$K and $p_C = 1$ atm as a function of its intrinsic, liquid-vapor contact angle θ_{LV} computed at $T = 300$ K, $p_C = 1$ atm. The error bars denote three standard errors.

Phase Equilibrium between Liquid Water or Ice and its Vapor If the radius R of the pore satisfies $R < R^{CV}_{crit}$ and the initial confined phase is vapor, the ambient condensed water should not impale or nucleate in the pore. Indeed, Figure S3 shows that ambient ice or liquid water initially resting on top of the surface texture will not fill the pore if the confinement length scale is satisfied.

The critical confinement length scales for the case in which ambient vapor outside the pore desublimates or condenses into the pore are highly sensitive to the vapor supersaturation. In Figure S4, typical water vapor supersaturation in a dense cloud under atmospheric conditions has been chosen as an illustrative case[3]; however, if the supersaturation is increased by 10%, the critical length scale decreases by around 99%.

Freezing and Melting Point Hysteresis Figure S5 demonstrates the hysteresis between the melting and freezing temperature that exists for hydrophilic substrates; for hydrophobic surfaces, the melting and freezing temperatures are equal. Figure 2 shows that the equilibrium state of the confined phase in the hydrophilic pore should be liquid water since $\theta_{IL} > 90$ for $\theta_{LV} < 90$; the confined ice is therefore metastable.

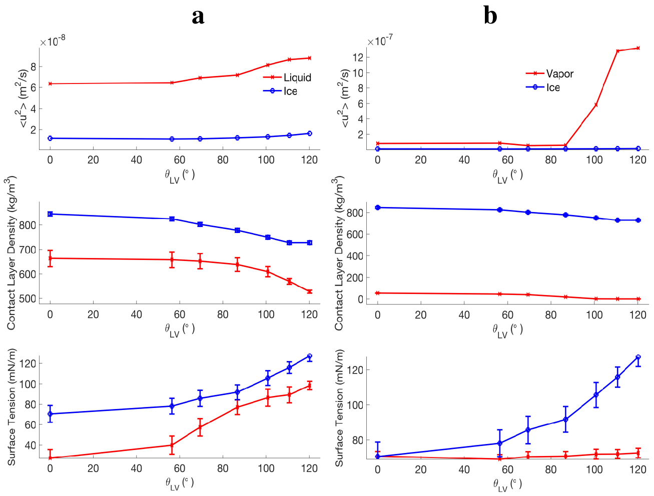

Figure S2. The mean squared displacement $< u^2 >$, density, and surface energy of (**a**) liquid water and ice as well as (**b**) vapor and ice in the contact layer (within 5 of the substrate surface) as a function of θ_{LV} (computed at $T = 300$ K and $p_C = 1$ atm). $T = 255$ K, $p_C = 1$ atm. The error bars denote three standard errors.

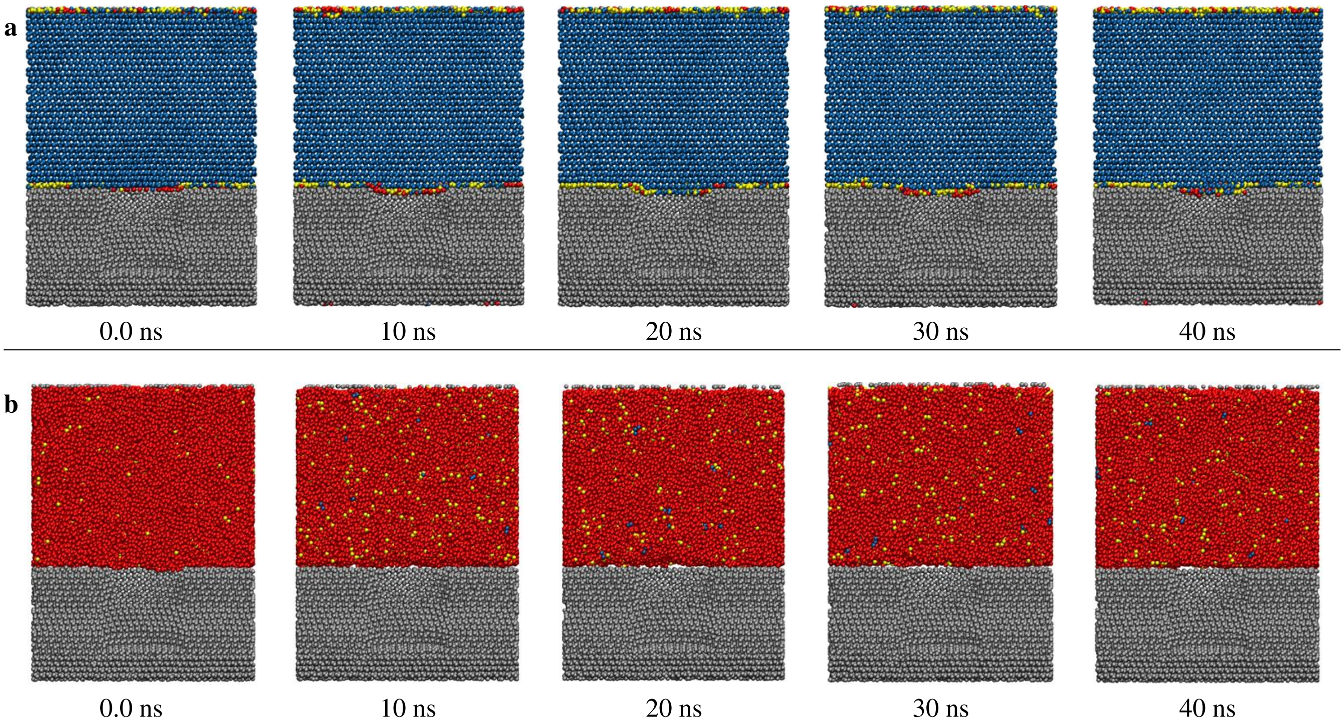

Figure S3. Molecular dynamics simulation of a 5 nm diameter cylindrical pore on a substrate with $\theta_{LV} = 120.2°$ (computed at $T = 300$ K) and $\theta_{IL} = 134.0°$ (computed at $T = 255$ K) at constant temperature 255 K, and pressure 1 atm. The height of the pore is 7 nm, and the radius of the pore $R = 2.5$ nm $< R^{CV}_{crit}$. Cross-sections of the simulations are presented in **a** and **b**. In trajectory **a**, the ambient ice (blue) rests on top of the surface (grey) initially and in the final equilibrium configuration. In trajectory **b**, the ambient liquid water (red) and amorphous glass (yellow) rests on top of the surface initially and in the final equilibrium configuration. Phases (lattice ice, amorphous glass, liquid water) were colour coded using the CHILL algorithm [2].

MD Verification of Critical Confinement Length Scale Figure S6 shows that the theoretically derived R^{IL}_{crit} as a function of ambient temperature matches simulation results. R^{IL}_{crit} clearly demarcates the transition zone that separates total liquid water occupation of the pore and total ice occupation. This transition zone describes intrusion of ambient ice into the pore via an ice-liquid meniscus that curves into the cavity.

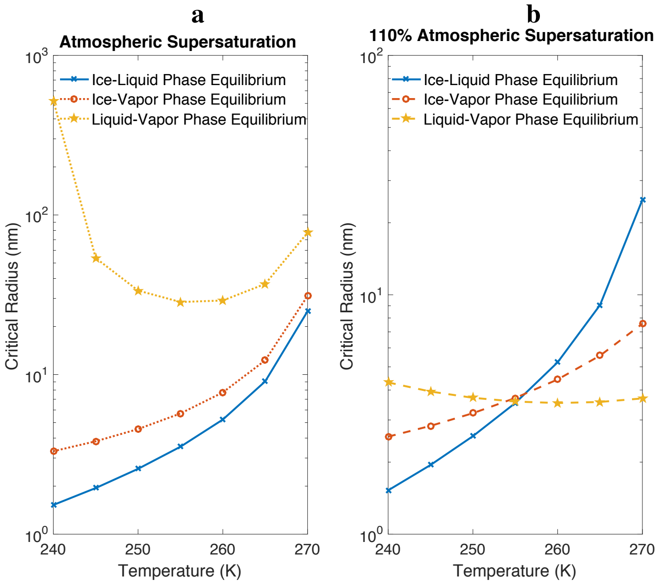

Figure S4. The critical pore radii for phase equilibrium between liquid water, ice or vapor as a function of temperature. For phase equilibrium between condensed water (liquid water, ice) and its vapor, the ambient phase outside the pore is vapor, with $p_V > p_S$. For **a**, the water vapor supersaturation is that typically found in dense clouds under atmospheric conditions[3]. In **b**, the vapor supersaturation is 110% that for **a**. The intrinsic contact angles of the substrate are taken to be constant: $\theta_{LV} = \theta_{IV} = \theta_{IL} = 120°$. This does hold not true in general for a specific substrate; however, a relationship between these contact angles has not been established experimentally or computationally in the literature. The assumption is not made for subsequent analyses.

Figure S7 uses molecular dynamics to find the largest pore radius for which the confined phase is liquid water, thereby estimating the critical confinement radius for the cylindrical pore as a function of substrate icephobicity θ_{IL}.

Table S1. The theoretical critical confinement length scale R_{crit}^{IL} compared to the largest pore radius for which the confined phase is liquid water, found from simulations (Fig. S7)

$\theta_{LV}(°)$ (computed at $T = 300$ K)	$\theta_{IL}(°)$ (computed at $T = 255$ K)	$\mathbf{R}_{crit}^{IL}$ (nm)	Simulation pore radius (nm)
120.2 ± 5.9	134.0 ± 11.3	3.8 ± 0.7	4
86.8 ± 8.0	110.6 ± 9.5	1.9 ± 0.8	2.5

Strength of Ice Adhesion The strength of ice adhesion is a practical engineering metric to gauge the anti-icing capabilities of engineered surfaces. Ambient ice in each system was sheared from the substrate at a constant velocity using a harmonic spring with spring constant K. One end of the spring was attached to the center of mass of the ambient ice $x_{com}(t)$. The other end was attached to a reference point $x_{ref}(t)$ at an equilibrium initial distance of $R = x_{spring}(0) - x_{com}(0)$ from the ambient ice. The reference point is displaced at a constant velocity v, such that $x_{ref}(t) = x_{ref}(0) + vt$. The force applied to the bulk ice in the shear direction is proportional to the spring deflection from the equilibrium length R.

$$F_{shear}(t) = K(x_{com}(t) - x_{ref}(t)) \tag{S8}$$

The maximum shear force at which ambient ice detaches from the surface can be recorded, and the strength of ice adhesion was taken to found by dividing the maximum shear force by the projected interface area.

The shear force was processed through a Gaussian filter to remove the high frequencies attributed to thermal fluctuations and the resonance frequency of the spring[4]. This is done to compare simulation results with experimental data, which often cannot resolve random thermal motion.

Pore Geometry Three typical pore geometries were tested for their ability to reduce the strength of ice adhesion: cylindrical, conical and triangular prisms. Figure S8 shows that the 60° angular constraints at the corners of the triangular prism

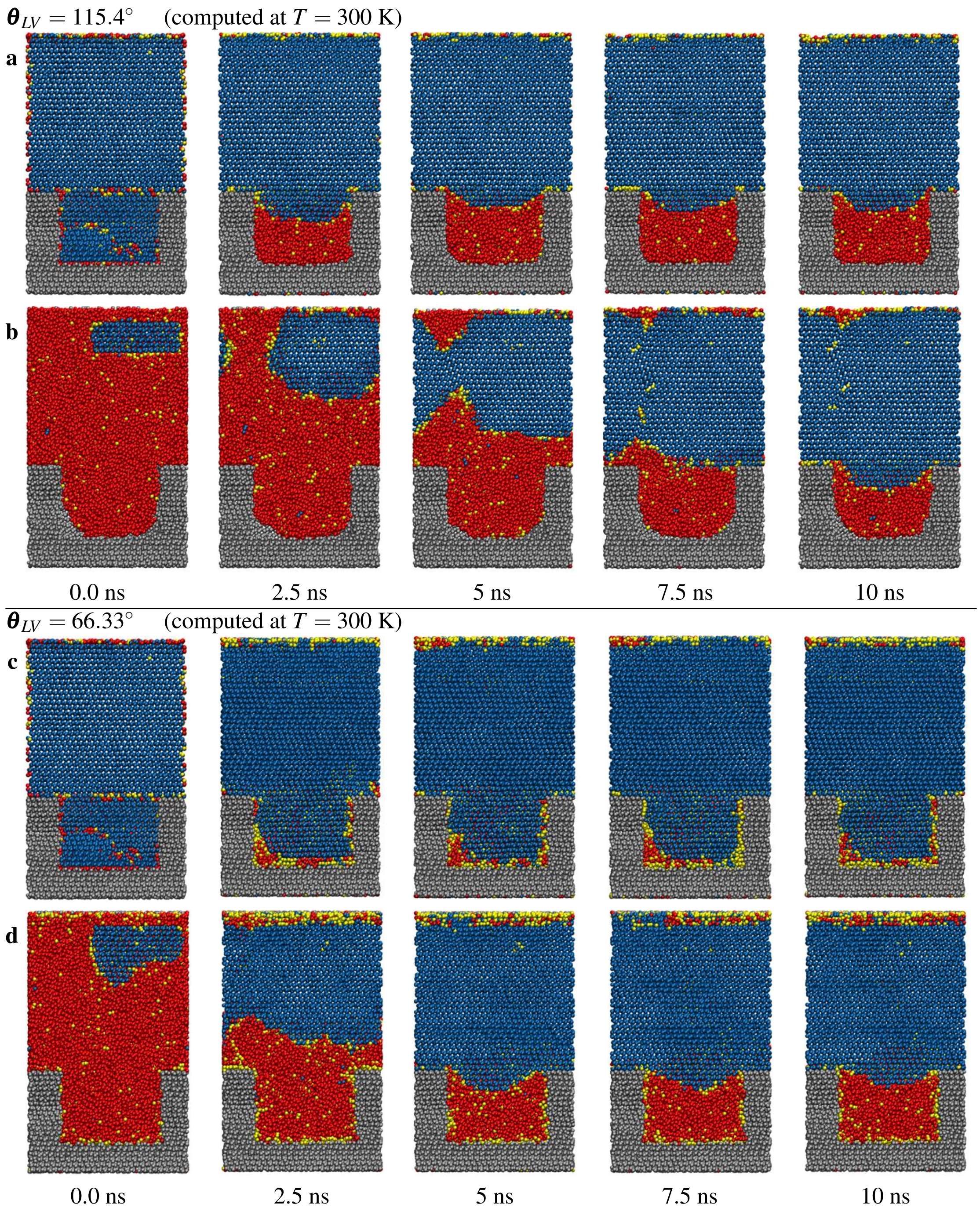

Figure S5. Molecular dynamics simulation of a 5 nm diameter cylindrical pore on a substrate with (**a**, **b**) $\theta_{LV} = 115.4^\circ$ (computed at $T = 300$ K) and with (**c**, **d**) $\theta_{LV} = 66.33^\circ$ (computed at $T = 300$ K). $T = 255$ K, and $p_C = 1$ atm. Cross-sections of the simulations are presented in **a**, **b**, **c** and **d**. In trajectories **a** and **c**, ambient ice (blue) is initially placed adjacent to a textured substrate (grey). For trajectory **a**, the ice melts passively inside the hydrophobic pore, producing amorphous glass (yellow) and liquid water (red) within the pore. For trajectory **c**, the ice does not melt in the hydrophilic pore. In trajectories **b** and **d**, liquid water is initially placed adjacent to a textured substrate, where an ice nucleus is artificially introduced to initiate growth of the ice phase. For both the hydrophobic and hydrophilic trajectories, the confined water does not freeze, instead remaining in the metastable liquid phase. Phases (lattice ice, amorphous glass, liquid) were colour coded using the CHILL algorithm[2].

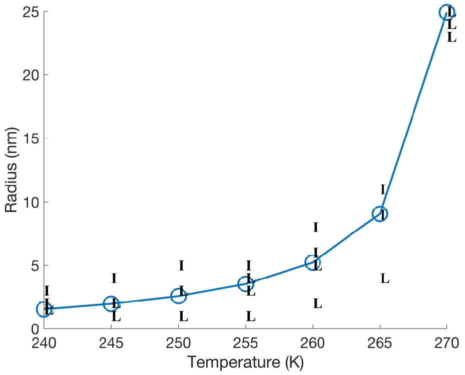

Figure S6. A comparison between molecular dynamics simulations and the theoretical critical confinement length scale. The blue line is the theoretical critical radii R^{IL}_{crit} calculated as a function of temperature; θ_{IL} is computed from equation (11) with $p_C = 1$ atm at different temperatures. The heights of pores are 7 nm. From molecular dynamics, the marker **L** indicates that the confined phase is predominantly liquid water (over 50% by mass inside the pore), whereas the marker **I** means the confined phase is predominantly ice. The liquid wettability of the substrate is $\theta_{LV} = 120.2°$ (computed at $T = 300$ K).

θ_{LV} = 120.2° (computed at $T = 300$ K)
θ_{IL} = 134.0° (computed at $T = 255$ K)

a

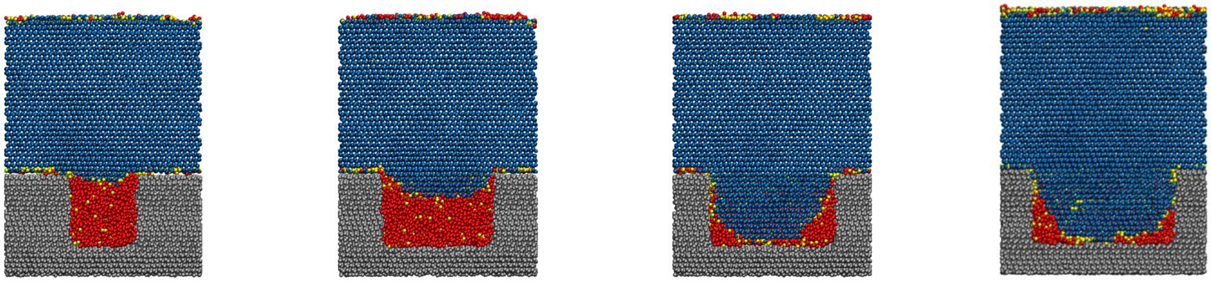

2.5 nm | 4.0 nm | 4.5 nm | 5.0 nm

θ_{LV} = 86.8° (computed at $T = 300$ K)
θ_{IL} = 110.6° (computed at $T = 255$ K)

b

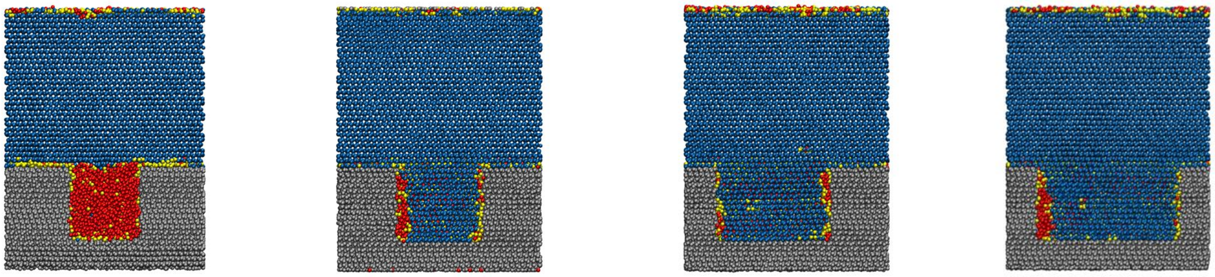

2.5 nm | 3.0 nm | 4.0 nm | 5.0 nm

Figure S7. Equilibrium states of water confined in a cylindrical pore on hydrophobic (**a**) and hydrophilic (**b**) surfaces, with radii ranging from 2.5 nm to 5.0 nm. Cross-sections of the simulations are presented. Liquid water is labeled red, ice is blue, amorphous glass is yellow, surface is grey. $T = 255$ K, and $p_C = 1$ atm.

makes the existence of ice unfavorable in the pore by by penalizing both cubic and hexagonal crystal lattices. This geometrical frustration results in a larger population of liquid water molecules in triangular pores than in other geometries. Triangular pores are therefore expected to reduce the strength of adhesion to a greater extent than cylindrical pores of the same projected surface area by decreasing the energy barrier between the local (attached ice) and global (detached ice) equilibria.

Conversely, the bottom of conical pores penalizes occupation by both the solid and liquid phase, such that fewer liquid water molecules are confined in the conical geometry; these pores are expected to be less effective than triangular and cylindrical

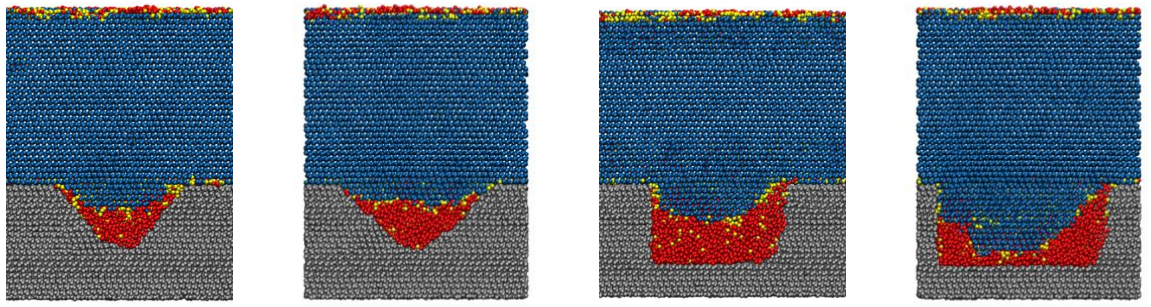

8 nm conical | 10 nm conical | 8 nm triangular prism | 10 nm triangular prism

Figure S8. Equilibrium states of various pore geometries on a substrate with $\theta_{LV} = 120.2^\circ$ (computed at $T = 300$ K), $\theta_{IL} = 134.0^\circ$ (computed at $T = 255$ K) at constant temperature 255 K, and pressure 1 atm. Cross-sections of the simulations are presented. The lengths refer to the radius of the circle occupying the same area as the projected top area of the pore. The height of the pores are 5 nm.

pores in reducing the strength of adhesion.

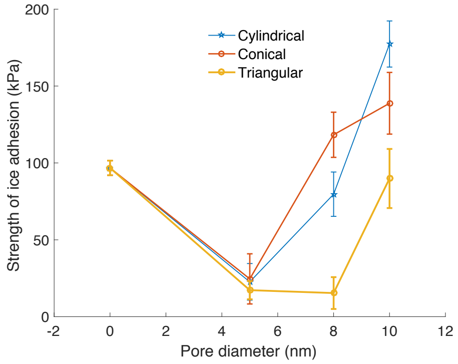

Figure S9. Strength of adhesion for various pore geometries. $T = 255$ K, $p_C = 1$ atm, $\theta_{LV} = 120.2^\circ$ (computed at $T = 300$ K), $\theta_{IL} = 134.0^\circ$ (computed at $T = 255$ K). The error bars denote three standard errors.

Fig S9 verifies the prediction that the strength of ice adhesion is largest for the conical pore and smallest for the triangular prism when liquid water predominantly occupies the pore. For substrates with pores mostly occupied by an ice phase ($R \geq R^{IL}_{crit}$), the strength of ice adhesion is largest for the cylindrical pore and smallest for the triangular prism. It is shown that triangular pores offer the most reduction in the strength of ice adhesion compared to that of a flat surface among the pore geometries compared.

Hierarchical Surface Texture Hierarchical roughness comprises small length-scale roughness superimposed over a large-scale roughness[5]. The different roughness scales may amplify the intrinsic ice-liquid contact angle θ_{IL} and increase the critical confinement length scale R^{IL}_{crit}. However, Figure S10 shows that although hierarchical roughness can induce the phase-change of confined water adjacent to the pore walls, the core confined phase away from the walls is largely unaffected. This suggests that superimposed roughness length scales do not significantly affect the critical roughness length scale governing the phase of the confined water.

Mechanism of Ice Shear The mechanism of ambient ice shearing from the substrate depends on the liquid wettability of the surface (Fig. S12). For a hydrophobic substrate with roughness satisfying $R \leq R^{IL}_{crit}$, the reduction in the strength of ice adhesion (Fig. 5) occurs due to the phase change of the confined, metastable liquid water to ice as the pore dewets during cleavage.

For a hydrophilic substrate with roughness satisfying $R \gtrapprox R^{IL}_{crit}$, phase change of the confined ice can also reduce the strength of ice adhesion to the substrate (Fig. S11). In this case, the confined ice does not escape the pore during cleavage. Instead, the shearing of the ambient ice induces melting of the confined ice near the walls of the pore. The resulting decrease in the surface energy ($\sigma_{SL} - \sigma_{SI} < 0$) reduces the strength of ice adhesion to a larger extent than the case where liquid water is sustained

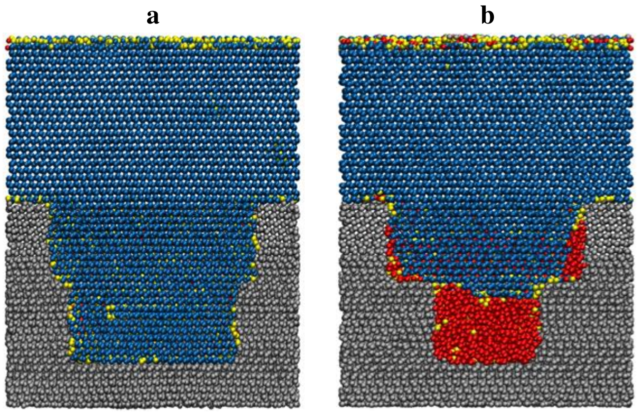

Figure S10. Equilibrium states of water confined in hierarchical surface texture. Cross-sections of the simulations are presented in **a** and **b**. (**a**) 9 nm diameter cylindrical pore at the bottom of a 11 nm diameter cylindrical pore and (**b**) 5 nm diameter cylindrical pore at the bottom of a 9 nm diameter cylindrical pore on a substrate with $\theta_{LV} = 120.2°$ (computed at $T = 300$ K), $\theta_{IL} = 134.0°$ (computed at $T = 255$ K), $T = 255$ K, P = 1 atm. The largest pore diameter for which the confined phase in a non-hierarchical pore is liquid water is 8 nm, as demonstrated by Figure S8.

inside the pore. This demonstrates that phase change of the confined water has a greater impact in reducing ice adhesion than passive lubrication effects.

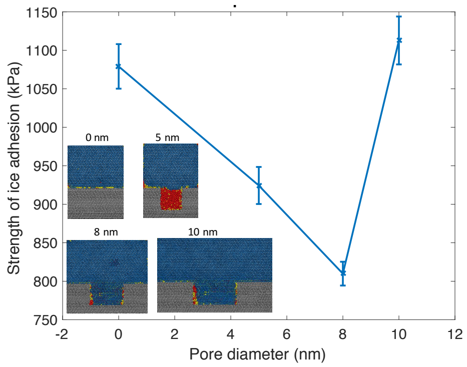

Figure S11. Strength of ice adhesion for cylindrical pores embedded in a hydrophilic substrate, compared with that for a flat surface (radius = 0 nm). $T = 255$ K, $p_C = 1$ atm, θ_{LV} = 86.8° (computed at $T = 300$ K), $\theta_{IL} = 110.6°$ K (computed at $T = 255$ K). The error bars denote three standard errors.

Periodic array of nanopillars The strength of adhesion for the periodic array of cylindrical nanopillars is shown numerically in Figure S13 as a function of the pillar radius and spacing (defined as the radius of the inscribed circle in the projected area between four adjacent pillars) for the simulated results. The contour plot in Figure 7 is interpolated from these values using a biharmonic spline.

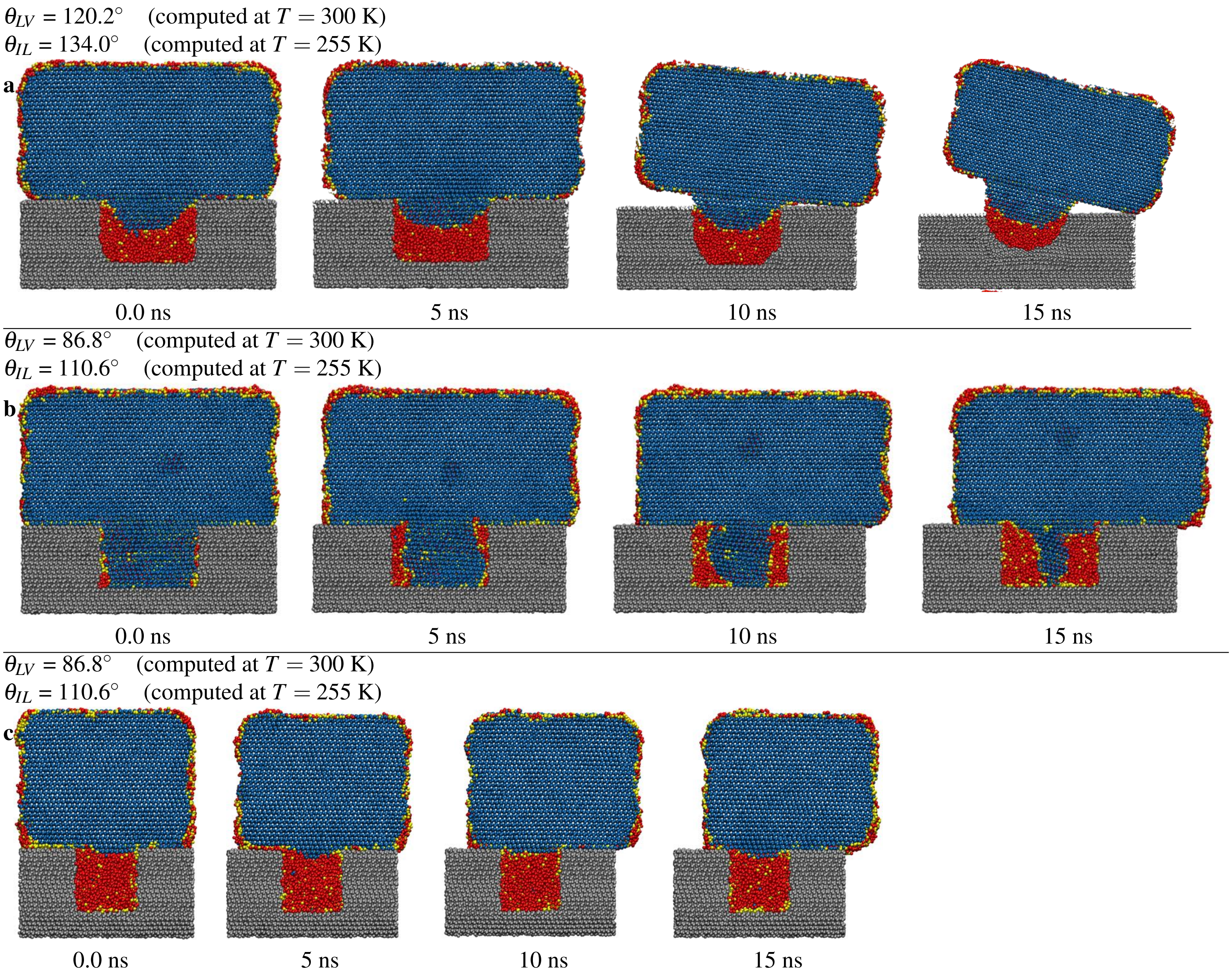

Figure S12. Molecular dynamics simulations demonstrate the fundamental difference between ice detachment pathways for (**a**) hydrophobic and (**b**, **c**) hydrophilic substrates. Cross-sections of the simulations are presented. $T = 255$ K, $p_C = 1$ atm and the pore diameters are (**a**, **b**) 8 nm and (**c**) 5 nm respectively. For hydrophobic substrates (**a**), the confined phase escapes during detachment, while for hydrophilic substrates (**b**, **c**), the confined phase is sustained within the texture under shear. For the hydrophilic substrate, the pore size therefore determines whether (**b**) phase change or (**c**) lubrication affects the strength of ice adhesion. Phases (lattice ice, amorphous glass, liquid) were colour coded using the CHILL algorithm[2].

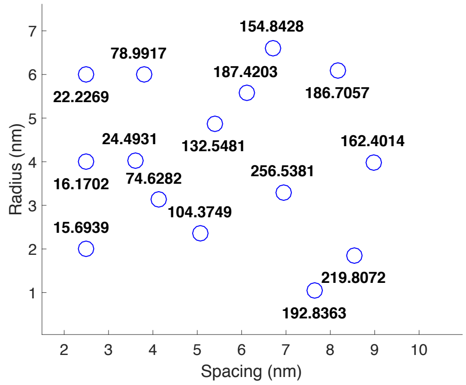

Figure S13. Strength of ice adhesion vs. radius and spacing (defined as the radius of the inscribed circle in the projected area between four adjacent pillars) of nanopillars in a periodic array, $T = 255$ K, $p_C = 1$ atm, $\theta_{LV} = 120.2°$ (computed at $T = 300$ K), $\theta_{IL} = 134.0°$ (computed at $T = 255$ K). The strength of ice adhesion for a flat surface is 96.75 kPa.

References

1. Alba-Simionesco, C. *et al.* Effects of confinement on freezing and melting. *J. Physics: Condens. Matter* **18**, R15 (2006). URL http://stacks.iop.org/0953-8984/18/i=6/a=R01.

2. Moore, E. B., de la Llave, E., Welke, K., Scherlis, D. A. & Molinero, V. Freezing, melting and structure of ice in a hydrophilic nanopore. *Phys. Chem. Chem. Phys.* **12**, 4124–4134 (2010). URL http://dx.doi.org/10.1039/B919724A. DOI 10.1039/B919724A.

3. Libbrecht, K. G. The physics of snow crystals. *Reports on Prog. Phys.* **68**, 855 (2005). URL http://stacks.iop.org/0034-4885/68/i=4/a=R03.

4. Marrink, S.-J., Berger, O., Tieleman, P. & Jähnig, F. Adhesion forces of lipids in a phospholipid membrane studied by molecular dynamics simulations. *Biophys. J.* **74**, 931 – 943 (1998). URL http://www.sciencedirect.com/science/article/pii/S0006349598740160. DOI http://dx.doi.org/10.1016/S0006-3495(98)74016-0.

5. Kwon, Y., Patankar, N., Choi, J. & Lee, J. Design of surface hierarchy for extreme hydrophobicity. *Langmuir* **25**, 6129–6136 (2009). URL http://dx.doi.org/10.1021/la803249t. DOI 10.1021/la803249t. PMID: 19466776, http://dx.doi.org/10.1021/la803249t.
